# The ethics of killing human/great-ape chimeras for their organs: a reply to Shaw et al.

**DOI:** 10.1007/s11019-015-9658-1

**Published:** 2015-08-21

**Authors:** César Palacios-González

**Affiliations:** Institute for Science Ethics and Innovation, The University of Manchester, Oxford Road M13 9PL Stopford Building, Room 3.383, Manchester, UK

**Keywords:** Chimeras, Human-nonhuman chimeras, Organ donation, Great ape, Nonhuman animals, Part-human

## Abstract

The aim of this paper is to critically examine David Shaw, Wybo Dondorp, and Guido de Wert’s arguments in favour of the procurement of human organs from human/nonhuman-primate chimeras, specifically from great-ape/human chimeras. My main claim is that their arguments fail and are in need of substantial revision. To prove this I first introduce the topic, and then reconstruct Shaw et al.’s position and arguments. Next, I show that Shaw et al.: (1) failed to properly apply the subsidiarity and proportionality principles; (2) neglected species overlapping cases in their ethical assessment; (3) ignored the ethics literature on borderline persons; and (4) misunderstood McMahan’s two-tiered moral theory. These mistakes render an important part of their conclusions either false or problematic to the point that they would no longer endorse them. Finally I will briefly mention a possible multipolar solution to the human organ shortage problem that would reduce the need for chimeras’ organs.

## Introduction

A chimera, in biological sciences, is an organism which at cellular level is composed from at least two different sets of cells, which originated in genetically diverse organisms. Chimeras can be divided in two broad groups: primary chimeras and secondary chimeras. “Primary chimæras are formed by mixing together two early embryos, or an early embryo with isolated embryonic cell types obtained from a different embryo or cultured stem cell line. The resulting chimæra has cells of different origins, in many tissues. Secondary chimæras are formed experimentally by transplanting (or grafting) cells or tissues into animals at later stages of development, including late fetal stages, post-natal or even adult animals. The donor cells are only present in a few tissues” (The Academy of Medical Sciences 2011, 18–19). It should be emphasised that the number, origin of the cells, and the timing of the mixing could produce very different outcomes in respect of the kinds of capacities a chimera could possess.

There are interspecific chimeras and intraspecific chimeras. Intraspecific chimeras are those where the sets of cells that make up the organism belong to *the same* biological species. For example, a human chimera can be created when two non-monozygotic early embryos fuse completely and grow into one body. The resulting entity is an organism that at cellular level is a ‘patchwork’ of both sets of human cells (Tippett 1983). Interspecific chimeras are those where the sets of cells that make up the organism belong to *different* biological species. For example, a goat/sheep chimera can be created by combining blastomeres from four-cell goat embryos with blastomeres from four-cell sheep embryos, the resulting entity is an organism that at cellular level is a ‘patchwork’ of goat cells and sheep cells (Fehilly et al. 1984).

Intraspecific nonhuman/nonhuman chimeras and interspecific human/nonhuman chimeras (henceforth, HNH-chimeras) are used both in biological research that aims at improving, or maintaining, human health and in research that aims at advancing our understanding of various biological mechanisms and processes. For example, mouse-human chimeras have been used in the research of human haematopoiesis; development and function of the immune system; infectious diseases; autoimmunity; cancer; human cell development, maturation and migration; and regenerative medicine (Shultz et al. 2007; Rashid et al. 2014; Sun et al. 2007; Tam and Rossant 2003; Lapidot 2001). HNH-chimeras have also been proposed as possible tools for creating vaccines against deadly diseases such as malaria, dengue, Hepatitis B, HIV and Hepatitis C (Davis and Stanley 2003; Sacci Jr. et al. 2006; Legrand et al. 2009; Bhan et al. 2010).

In recent work David Shaw, Wybo Dondorp, Niels Geijsen, and Guido de Wert have examined, from an ethical and legal position, the procurement, for human transplantation purposes, of human organs created within HNH-chimeras. In one paper Shaw examined the legal status of creating HNH-chimeras for obtaining human organs under Swiss legislation; in another, Shaw, Dondorp, Geijsen, and de Wert assessed the ethical issues surrounding the procurement of human organs from human/pig chimeras; and in a final paper Shaw, Dondorp, and de Wert assessed the ethical issues surrounding the procurement of human organs obtained from human/nonhuman-primate chimeras (Shaw 2014; Shaw et al. 2014a, b). This philosophical research stems, in part, from recent advances in induced pluripotent stem cell (iPSC) research, and the ad hoc creation of solid organs within intraspecific and interspecific chimeras (Kobayashi et al. 2010; Isotani et al. 2011; Usui et al. 2012; Matsunari et al. 2013; Kobayashi et al. 2014; Rashid et al. 2014).

The aim of this paper is to critically examine the arguments that have been advanced by David Shaw, Wybo Dondorp, and Guido de Wert in favour of the procurement of human organs obtained from human/nonhuman-primate chimeras, specifically from *great*-*ape/human chimeras*. There are two important reasons for examining such arguments. First, for those that hold that there are degrees of moral status, based upon cognitive capacities,^1^ the ethics of using great apes for medical research that aims at benefiting humans is a topic that merits special attention. Second, the ethics of using chimeras for their human organs is a topic that warrants notice in the medical ethics literature at this point given recent developments in biological sciences. For example, in a recent paper Madhusudana Girija Sanal has sketched a road map for how to create a transplantable human liver within a chimpanzee/human chimera (Sanal 2011).

My claim is that the arguments that these authors have advanced fail to support their conclusions, and thus they are in need of substantial revision. In order to prove this first I present a detailed reconstruction of Shaw et al.’s arguments. Secondly, and relying on a cognitive capacities account of moral status, I show that Shaw et al. committed major errors: (1) not properly applying the subsidiarity and proportionality principles; (2) not including species overlapping cases in their ethical assessment; (3) ignoring the ethics literature on borderline persons; and (4) misunderstanding McMahan’s two-tiered moral theory. Finally I will mention a possible multipolar solution to the human organ shortage problem that would reduce the need for HNH-chimeras’ organs.

## Human organs, nonhuman animal research, and moral status

Shaw et al. start their inquiry, in *Using Non*-*Human Primates to Benefit Humans: Research and Organ Transplantation*, by bringing forward a well-known fact about the current status of human organ transplantation: there is a scarcity of human organs. This in turn means two things: (1) that many people that are currently affected by fatal illnesses that can only be treated by organ transplantation—to a full or less extent—will die waiting for an organ, and (2) that many people in need of an organ will experience pain and suffering while waiting for it. For example, in the UK three people die every day due to the shortage of transplantable organs; and in United States thirty people die every day, or are removed from the waiting list due to being too ill to receive a transplant (Humphreys 2014; NHS 2014).

There are several courses of action that can be taken in order to increase the number of human organs available for transplantation: (1) changing from an opt-in donation system to a default opt-out donation system, (2) creating incentives for people to donate their organs after their death, (3) creating incentives for people to make live organ donations, (4) making after-death organ donation obligatory, and (5) creating educational campaigns to increase the number of organ donations. In addition to these proposals there are five alternative strategies, which do not require ‘human’ donation as such: (6) the creation of in vitro organs using scaffolds, (7) 3-D printing of human organs, (8) xenotransplantation, (9) the creation of mechanical organs and (10) *chimera based organ transplantation*.

As I have previously stated, Shaw et al. decided to explore the last possibility in part because of recent scientific breakthroughs in chimera research. Of all the possible kinds of HNH-chimeras that could be created for obtaining human organs they focus on two: pig/human chimeras and human/nonhuman-primate chimeras.^2^ Their selection is not arbitrary: those chimeras are considered to be the best possible biological candidates for the creation of transplantable human organs.

Before moving forwards two things should be made clear about the current state of this research. First, the creation of *human* solid organs within HNH-chimeras has not been accomplished.^3^ Second, there are still substantial technical hurdles that must be overcome in order for the creation of transplantable solid human organs to be feasible. Given these facts Shaw et al.’s discussion assumes, *ex hypothesis*, that this research will be successful and thus that the technical hurdles will be overcome. Assuming the former evades issues of risk and harm, due to imperfect research, but it allows them to focus on the morality of *killing* HNH-chimeras for their human organs. Throughout the rest of the paper I will also assume that this research will be successful.

After acknowledging that human organs for transplantation are a scarce resource, and assuming that human organs from chimeras will be available, Shaw et al. turn their attention to animal research. They point out that nonhuman animal research, for human health purposes, is carried out all around the world; and that the present day moral justification for doing so is that its aims (i.e. the improvement and maintenance of people’s health) are so important that, in principle, they trump over all nonhuman animals’ interests (e.g. avoiding pain, being comfortable, staying alive). Two caveats should be mentioned. First, and I will return to this point in the section “The Case Against Killing Great-Ape/Human Chimeras for Their Organs”, there are morally relevant distinctions between killing certain animals and killing others. Second, this is not to say that all medical research that employs nonhuman animals can be morally justified.

When questioned about *how* nonhuman animal research should be carried out Shaw et al. appeal to the well-established principle of ‘3 Rs’ (replacement, reduction and refinement) which, according to them, in turn is grounded in two other principles. “The 3 Rs are in turn based on two key principles: proportionality and subsidiarity. These state, respectively, that any use of animals for research must be proportional to the prospective benefit, and that animals should only be used when no reasonable alternative is available” (Shaw et al. 2014a, 573).

The normative force of the proportionality and subsidiarity principles, when applied to nonhuman animal research, in turn relies, at least in part, on a theory of moral status. Such a theory should be able to explain why it is morally distinct to use, for research purposes, certain nonhuman animals rather than normal adult human beings. In their paper Shaw et al. do not *explicitly* endorse a particular theory of moral status, but it can be inferred that they are adopting a *non*-*speciesist cognitive capacities account*. This is supported by the fact that they reject an anthropocentric account of moral status (being a member of the *Homo sapiens* species is a necessary or sufficient condition for possessing moral status), that they accept that certain nonhuman animals possess moral status (sentience being a sufficient condition for possessing it, given that it is a sufficient condition for possessing experiential welfare), and also that they appear to align themselves (Shaw et al. 2014a, 574) with Singer’s definition of personhood [a person is a “rational and self-conscious being” (Singer 1993, 87)]. If this is correct then it is safe to assume that they would accept that the creatures with most moral worth are persons, after them sentient beings and lastly (and if possible) non-sentient creatures.

### Border-line cases and animal experimentation

Shaw et al. maintain that even when a cognitive capacities account of moral status can be used to determine the moral permissibility of most nonhuman animal research there are ‘border-line cases’ where it *cannot* provide a ‘clear-cut’ answer. For example, is it morally permissible to carry on painful and destructive research in a nonhuman primate that has the relevant psychological capacities to a slightly lower degree than a human person? Confronted with such ‘border-line cases’ Shaw et al. quote McMahan’s ‘Time-Relative Interest Account’ as a *possible alternative explanation* of why harming nonhuman animals with *slightly less* psychological capacities than human persons is less objectionable than harming human persons. They cite the following paragraph:

The Time-Relative Interest Account offers an explanation of why the killing of animals is less seriously objectionable than the killing of persons. Because the psychological capacities of animals are significantly less well developed than those of persons, the range of goods accessible to them is narrower and the degree of psychological unity within their lives is less. They therefore have a weaker time-relative interest in continuing to live than a person normally does. (McMahan 2003, 204)

After citing this paragraph, Shaw et al. assert that the *sliding scale* nature of McMahan’s Time-Relative Interest Account also *cannot* tell us if the use, for research purposes, of nonhuman animals with *slightly less psychological capacities* than human persons is morally justified. Therefore, for them, a cognitive capacities account of moral status and McMahan’s Time-Relative Interest Account are unable to show where the ‘cut-off’ between morally permissible and impermissible research should be (Shaw et al. 2014a, 574). This ‘cut-off’ is of paramount prominence for Shaw et al.’s discussion about killing human/nonhuman-primate chimeras for their organs, because, as they accept, some nonhuman primates in fact possess only slightly fewer psychological capacities than human persons (the most prominent example being great apes) and thus belong to the ‘grey area’ between persons and non-persons. Finally, it is important to emphasise, for reasons that will become clear afterwards, that Shaw et al. *only* cite McMahan’s previous paragraph.

Once they have pointed out the supposed *sliding scale* problem of both accounts they move on and claim that the consensus on the use of nonhuman primates—which, depending on the species, may or may not be in a personhood ‘grey area’—for research that benefits human health is that their use is *necessary and justified* (Shaw et al. 2014a, 575). The *scientificrationale* behind this is that nonhuman primates are biologically the most similar to humans and they offer the most accurate nonhuman animal model.

Even when a scientific rationale is provided, Shaw et al. need to further morally justify the use, for research purposes, of nonhuman primates with slightly fewer psychological capacities than human persons (taking for granted that, in principle, it is morally permissible to sacrifice sentient beings for the sake of persons). They need to do so because according to them neither a cognitive capacities account of moral status nor the Time-Relative Interest Account were able to tell us if using such nonhuman animals for research is morally permissible. The way in which Shaw et al. provide the needed moral justification is by invoking the subsidiarity and proportionality principles. They assert: “Given that the only alternative would be to use humans, the subsidiarity criterion [for using nonhuman primates for research] is met, and given the substantial potential benefits of some of the treatments that may result, the proportionality test is also met” (Shaw et al. 2014a, 574).

It is important to point out that the authors realise that if they accept a cognitive capacities account of moral status then the claim that *all* nonhuman primates have less moral value than *all* human beings is in fact incorrect. This is why they emphasise that “to the extent that primates actually meet the criteria for personhood they should be treated as persons rather than animals, making the proportionality and subsidiarity principles irrelevant” (Shaw et al. 2014a, 574). Shaw et al. then stress that they “assume that the current consensus position is correct, and primates (*with the possible exception of great apes*) [emphasis added] are not persons [and thus can be used for certain research purposes]” (Shaw et al. 2014a, 574).

A problem arises here: Shaw et al. do not take into account, for their ethical assessment, the fact that *there are humans that are not persons*, and thus they do not distinguish throughout their text between human persons and human nonpersons. In the section ‘The Case Against Killing Great-Ape/Human Chimeras for Their Organs’ I will show why this important omission has serious repercussions for their arguments.

### The case for killing human/pig chimeras for their organs

Once they have examined whether it is morally permissible to use nonhuman primates for research that benefits human health and found that with the *possible* exception of great apes it is justified according to the proportionality and subsidiarity principles, Shaw et al. query if it would be morally justified to kill human/pig chimeras for their human organs. For them the question is easy to answer: yes. If we accept, they claim, that the use of certain nonhuman animals for destructive research is morally justified when the aims are those of saving human lives or improving human health, then, by a matter of consistency, we would also have to accept that, in principle, the use of human/pig chimeras as organ suppliers is morally justified. This justification works as long as human lives are saved or improved by such organs to the same extent that nonhuman animal research improves and saves human lives (and taking for granted that such chimeras are not persons).

Shaw et al. also argue that contrary to nonhuman animal research, that can lead to dead-ends, produce only indirect benefits, or produce results that are useless for human health improvement (due to the translational gap between nonhuman animal research and human research), the use of human organs that had their origin in human/pig chimeras would in most cases *save* at least one human life. This point is made to reinforce the position that killing human/pig chimeras for their organs is morally less problematic than most destructive nonhuman animal research.

While for these authors sacrificing a human/pig chimera in order to *save* a human life seems morally unproblematic, it appears that cases where its killing would *only* bring an improvement to someone’s well-being (e.g. reducing the suffering of dialysis) needs further justification, which Shaw et al. do not provide. This justification is required because the fundamental interests of the human/pig chimera must be weighed against the human’s interest of not suffering gravely. In this respect McMahan’s Time-Relative Interest Account may provide the needed justification. According to it, the ‘badness’ of death is a function of the lost opportunities of worthwhile life (however this is defined: preference satisfaction, pleasure, etc.), considered from a whole-lifetime perspective but proportionally adjusted to the strength of one’s psychological unity over time—the more psychological unity over time one has the more death harms one. If this is the case, we can say that a human person’s interest in not greatly suffering would trump over the human/pig chimera’s interest in staying alive if the human person’s time-relative interest in not suffering is weightier than the chimera’s time-relative interest in remaining alive. While is difficult to precisely calculate the amount of pain a person should be experiencing for it to be morally permissible to kill such chimera, I think that a *human person’s great suffering* would justify the painless killing of a human/pig chimera.

While the sacrifice of human/pig chimeras for the procuration of human organs for saving or lessening great suffering in human persons seems morally justifiable, the morality of procuring human organs from human/nonhuman-primates needs further elaboration.

### The case for killing human/nonhuman-primates chimeras for their organs

In order to investigate the morality of killing human/nonhuman-primate chimeras for their organs, Shaw et al. test whether such cases meet the proportionality and subsidiarity principles. They take this course of action because, as stated previously, they consider that a cognitive capacities account of moral status and McMahan’s Time-Relative Interest Account cannot provided a ‘clear-cut’ answer to the question of whether it is morally permissible to kill beings that possesses *slightly less* mental capacities than humans.

For them, killing human/nonhuman-primate chimeras for their organs *is proportional* given the potential direct benefits that would be conferred to humans.^4^ Such benefits are not only measured in the number of saved lives, but also in the reduction of suffering due to the shortening of waiting time that elapses between someone needing an organ, and her receiving it. Once they have accepted this they wonder if the distinction between greater and lesser apes is morally relevant:

The fact that *great apes* [emphasis added] might be necessary due for organ creation also raises the question of whether the distinction between lesser and great apes is relevant in this context. Most primates used in research, and all used in the UK, are smaller primates. While these could be used for organ production (especially for children), *bigger primates are more likely to be appropriate donors* [emphasis added]. (…) While the creation of human organs inside primates appears to meet the proportionality criterion *(with the possible exception of great apes)* [emphasis added], the subsidiarity principle raises further questions. (Shaw et al. 2014a, 576)

Justifying the killing of human/nonhuman-primate chimeras for their human organs, in terms of the subsidiarity principle, is more challenging because it must be shown that there are no reasonable alternatives for obtaining the much needed organs. To do this Shaw et al. first identify two possible readings of the subsidiarity principle, a strict reading and a permissive reading, and then they investigate their normative force. The strict reading asserts that if there is a reasonable alternative to the use of chimeras then we should not use them. The permissive reading affirms that using chimeras is permissible if non-problematic alternatives have not been established.

Once they have identified these two readings, Shaw et al. argue that even when the strict reading is accurate, in the description of the states of the world, it lacks normative force for the people who will die without an organ transplantation. It lacks such force because it does not follow from the fact that there are other reasonable possible courses of action that they can in fact be followed: “for the specific people who will die soon without an organ there really is no alternative, suggesting that the restrictive reading of the subsidiarity principle is too strict” (Shaw et al. 2014a, 576). Given this Shaw et al. conclude that the *permissive reading* of the subsidiarity principle should be embraced. Therefore killing human/nonhuman-primate chimeras for their organs is morally justifiable, because there is *no real* reasonable non-problematic alternative available.

At this point Shaw et al. make a clarification in respect to what they think are *justifiable aims* for using human/nonhuman-primate chimeras’ human organs: “it could also be argued that primates should only be sacrificed to create organs when it’s necessary to *save life* [emphasis added] rather than to improve quality of life. While lessening the suffering of dialysis for kidney patients, for example, is a good goal, it is not clear that it is worth sacrificing a great ape for; in contrast, *such a sacrifice seems more appropriate if a human will die without it* [emphasis added]” (Shaw et al. 2014a, 577).

There are two ideas here that merit unpacking. The first is that human/nonhuman-primate chimeras should only be killed when otherwise a human would die. In such an assertion it is implied that human/nonhuman-primate chimeras’ time-relative interest in staying alive trumps over the human’s time-interest in not suffering. Two things should be further noted here. First, these authors do not address whether a specific degree of suffering (e.g. unbearable suffering) would merit killing human/nonhuman-primate chimeras. Second, they also do not address the question of whether human/nonhuman-primate chimeras should be killed for the sake of the health of human nonpersons, or humans with the same mental capacities as those of the chimeras providing the organs.

The second idea is that for Shaw et al. *it would be morally justifiable* to sacrifice a great-ape/human chimera for procuring human organs when a human would die without them. It is important to highlight that even when Shaw et al. had previously accepted that it is possible that great apes, and in this case great-ape/human chimeras, are in fact persons they have now asserted that *they are sacrificable* when doing so would save *any* human live. The idea that great apes’ lives are sacrificable when the life of a human is at stake is a common one, as McMahan has pointed out: “Most people believe that it would be permissible, and perhaps morally required, to kill an adult chimpanzee if the transplantation of its organs could save the life of an adult human being” (McMahan 2009, 584).

To sum up, Shaw et al. defended that killing human/nonhuman-primate chimeras for their organs would only be morally justified when such actions in fact would save a human’s life. They defended this by employing the proportionality and subsidiarity principles, while noting that neither a cognitive capacities account of moral status nor McMahan’s Time-Relative Interest Account could provide a ‘clear-cut’ answer to the morality of killing human/nonhuman-primate chimeras for their organs. This conclusion also applies, *in terms of the subsidiarity principle*, to cases of killing great-ape/human chimeras for their human organs.

## The case against killing great-ape/human chimeras for their organs

Shaw et al. have argued that the subsidiarity principle would *allow* killing great-ape/human chimeras for their organs when doing so would save a human’s life, and they have left open to further investigation (with the phrase ‘with the *possible* exception of great apes’) whether the proportionality principle would also allow for such killing. In this section I will show two things: (1) contrary to what Shaw et al. assert the subsidiarity and proportionality principles in fact *would not allow* killing great-ape/human chimeras for their organs, and (2) defending the killing of human/nonhuman-primate chimeras for their organs is more complicated than they appreciate.

It could be argued at this point that the interpretation I am offering of Shaw et al.’s position is uncharitable, given that they *do not explicitly endorse* the killing of great-ape/human chimeras for their organs. To this possible rejoinder I offer two rebuttals. The first is that Shaw et al.’s paper discusses the killing of creatures with slightly less psychological capacities than human persons, which is how we would define great apes. The second is that Shaw et al. accept that there are proper motives for killing a great ape (e.g. saving a human life) and that there are other inappropriate motives for doing so (e.g. increasing someone’s quality of life).

### Revisiting moral status

To understand where the problem lies with Shaw et al.’s conclusion we need to remember that they appealed to the subsidiarity and proportionality principles after they ‘found’ that a cognitive capacities account of moral status and McMahan’s Time-Relative Interest Account could not tell us, in a ‘clear-cut’ fashion, if it would be morally permissible to kill human/nonhuman-primate chimeras (including great-ape/human chimeras) for their human organs. In order to clear things up we need to go back to the discussion about moral status.

To state that an entity possesses moral status is to realise that “*in its own right and for its own sake, it can give us reason to do things such as not destroy it or help it* [emphasis in the original]” (Kamm 2007, 229). Such action guiding reasons are based, at least partly, on the entities’ interests, or time-relative interests. This in turn means that our consideration of an entity that possesses moral status should not be primarily conceived in terms of indirect duties towards ourselves, or duties towards other moral agents.

Among the beings with moral status there is a subset that we call persons. Persons are considered to be a distinct set of beings based on the specific cluster of capacities that they possess. The capacities that are commonly associated with personhood are: autonomy, rationality, self-awareness, linguistic competence, sociability, moral agency, and the capacity for intentional action. Providing a comprehensive list of necessary and sufficient capacities for personhood is unfeasible, because of the way in which they relate with each other. As DeGrazia has pointed out, someone is a person when she possesses the aforementioned capacities in a clustered way, not analysable in terms of one specific subset, to the point that suffices for a complex form of consciousness to be. To be a person one needs to possess enough of these capacities “where ‘enough’ takes account both of how many of these properties are instantiated and of the degree to which each is instantiated” (DeGrazia 2007, 320). Someone that is capable of normative self-governance (i.e. that is capable of reflecting upon her beliefs and actions and changing them accordingly) is taken to be a *paradigmatic person* under a cognitive capacities account of personhood.

The distinction between persons and nonpersons becomes relevant when they have conflicting interests. For example, in the transplantation cases that we have been discussing (i.e. the human/pig chimera cases), the interest that a person possesses in remaining alive is in direct conflict with the interest that a nonperson chimera has in remaining alive. One way of dealing with such conflict of interests is to weigh them according to the Time-Relative Interest Account and to resolve in favour of that that possesses stronger interests. If we did so we would have to resolve the conflict in favour of the person, because death deprives her of a broader range of goods than those that the nonperson would be deprived of.^5^

It is important to bear in mind that having moral status or being a person is species neutral. This means that from a normative stand point it should be regarded as irrelevant to which biological species an entity belongs when assessing if it possesses, or not, moral status, and if it is, or is not, a person. It is true that there are biological species whose members regularly possess some cluster of these capacities, but this does not mean that there cannot be members of this species that lack enough of the capacities to have moral status or to be persons (e.g. human foetuses or the congenitally severely cognitively impaired). This clarification about moral status and personhood is important because until recent times it was considered that all humans had more moral worth than *all* nonhuman animals and that *only* humans had the requisite capacities for personhood.

### The moral status of great apes

The question that we need to answer now is if great apes are persons, and thus if the strength of their interests is the same as those of human persons. Before advancing any further let’s remember that when we talk about great apes we are talking specifically about chimpanzees, bonobos, gorillas, and orang-utans. There are really strong indications that great apes in fact possess many of the characteristics that we associate with personhood.

Among the empirical evidence that we possess for asserting that great apes possess many personhood-relevant properties^6^ is the following: chimpanzees use tools such as stones to crack nuts, sticks to reach for insects, and moss for sponges. All great apes perform intentional actions and participate in social manipulation. All of them are bodily self-aware, and this self-awareness is revealed prominently in their imitation of bodily gestures and use of televised images of their out-of-view arms to reach concealed objects. They are capable of using mirrors to inspect otherwise imperceptible markings. Great apes also have social structures and they establish long-term relationships, dominance hierarchies, and allegiances. They shift allegiances, have knowledge of their position within a group, and expectations of others’ behaviours in accordance with their position in the group. All great apes have a rudimentary culture, they transmit, from one generation to another, novel behaviours such as knowledge of which leaves can be used for certain medicinal purposes, how to create certain types of tools and how to construct nests. Furthermore, there is evidence of proto-moral agency among certain great apes, the strongest of which is altruistic actions that do not appear either to be conditioned or instinctive. Finally there is at least one bonobo, one gorilla and one orang-utan that after intensive human language training were able to acquire and understand, to some extent, human spoken and sign language.

Now, even when great apes possess all these capacities they are not as linguistically competent as normal adult humans are, and they are not as capable of normative self-governance as normal adult humans are. This being the case, and under a strict interpretation of what personhood is, we should agree that great apes *are not* paradigmatic persons.^7^ Even when this is so we should acknowledge that great apes are *borderline persons*, just as David DeGrazia has forcefully and satisfactorily defended (DeGrazia 1997, 2005, 2007). What does it mean to say that great apes are borderline persons? It means, in the first place, that great apes inhabit the grey area between paradigmatic persons and nonpersons. They are beings with *slightly fewer psychological capacities than normal humans*. It is important, at this point, to remember that for Shaw et al. it is *only if* primates meet the criteria for personhood that the proportionality and subsidiarity principles should be regarded as irrelevant (Shaw et al. 2014a, 574). This further affirms that for them the proportionality principle in fact would allow for the killing of great-ape/human chimeras for their human organs, just as the subsidiarity principle does.

Before moving on we need to address the more general question of how moral respect relates to possessed cognitive capacities. If we accept, just as Shaw et al. seem to do, that moral status follows from certain cognitive capacities and that in the natural world beings exist within a continuum of cognitive capacities (that go from none at all to personhood related capacities) then we have to accept either: (1) that moral consideration should be proportional to the capacities a being possesses, or (2) that there is a threshold above which all beings must be treated alike. One obvious difficulty with a proportional respect approach (in contrast with a threshold one) is that it accepts differences in moral worth between persons. For example, because on this approach moral worth follows from the possession of more sophisticated capacities, seven year old normal human children, who have less sophisticated cognitive capacities than other persons such as eighteen year old normal humans, could be considered as having less moral worth. This being the case a two-tiered morality account, one for persons and the other one for nonpersons, seems more appropriate (Harris 1985).

In point of fact McMahan, in *The Ethics of Killing*, develops and endorses a two-tier account of morality! According to it a morality of *identical respect* must hold among persons, making their killing *equally prima facie wrong*, while for nonpersons the Time-Relative Interest Account should prevail. As he says, when discussing the morality of late term abortion:

It is important to remember that the Time-Relative Interest Account is not a complete account of the morality of killing, but is instead just one component of the more comprehensive Two-Tiered Account. According to the Two-Tiered Account, the wrongness of killing beings who are above the threshold of respect is governed by a requirement of respect. Thus even if a person has a very weak time-relative interest in continuing to live because the amount of good in prospect for him is quite small, it would nevertheless be just as wrong to kill him as it would be to kill any other person, if other things are equal. For his worth as a person is a function of his intrinsic nature and is unaffected by the amount of good the future holds in prospect. It is only in the case of beings that fall below the threshold of respect that the morality of killing is governed by the Time-Relative Interest Account. (McMahan 2003, 276)

The former quote highlights one of the fundamental problems with Shaw et al.’s paper: by only citing one paragraph of McMahan’s position, and not contextualizing it, they present a *deeply incorrect* version of McMahan’s moral theory. In actuality McMahan’s two-tiered account is completely capable of accommodating cases of animals with slightly less psychological capacities than normal human adults, as will become clear in the next section.

### The morality of killing borderline persons

So far I have only stated that great apes and great-ape/human chimeras are borderline persons. I now need to present a convincing case for the following claim: great apes and great-ape/human chimeras should be treated as paradigmatic persons. The justification for treating such creatures as paradigmatic persons is that the gap between the strength of the interests of persons and the strength of the interests of borderline persons is so small, and blurred, that they should be treated alike. This is a level-up solution to the ‘problem’ of borderline persons. As DeGrazia has stated:

[O]n any reasonable model of moral status [Unequal Considerations Model or Unequal Interests Model] borderline persons – whether human or nonhuman – have full moral status, or (though this may come to the same) ought to be regarded as having it. To treat borderline persons accordingly is to regard them much as we regard human children: not as substantially autonomous or as having full-fledged moral agency but as deserving moral protections of full strength. (DeGrazia 2007, 323)

The conclusion, if we adopt a level-up stance, is that it, *in principle*, is morally impermissible to kill human or nonhuman borderline persons for their organs. Thus: *it is morally impermissible to kill great apes and great*-*ape/human chimeras for their organs*.

A possible rejoinder which Shaw et al. could advance, is that in fact borderline persons should be treated according to McMahan’s Time-Relative Interest Account. They could try to justify this point by stressing that borderline persons’ interests should in fact be treated with slightly less consideration than those of persons, but that that slight difference allows for their killing. This slight difference explains why it would be less seriously objectionable to kill borderlines persons (e.g. great-ape/human chimeras) than to kill human persons. If Shaw et al. decided to embrace this position they would be opting for a level-down solution to the ‘problem’ of borderline persons. While this position might appear sensible, it has some logical implications that Shaw et al. should address in order for their case to be regarded as plausible.

The first implication, from a species neutral perspective on moral status and personhood, of such a level-down stance is that this would also mean that *all creatures* with the same, or less, psychological capacities than great apes could be sacrificed for their organs and this would be morally justifiable. Thus *all humans* that have the same, or less, psychological capacities than great apes can be morally sacrificed for their organs. Among the humans that could therefore be morally sacrificed are infants, toddlers, and adults that have less or the same mental capacities than great apes. Even if Shaw et al. claimed, and we agreed, that infants and toddlers should not be sacrificed for their organs because they have the *potential* to become paradigmatic persons, they would have to accept that there are two sets of cases where humans could be morally sacrificed for their organs. Cases where a human did not have the potential to become a paradigmatic or borderline person, and cases where they only had the potential to develop to a stage analogous to that of a great ape. For example, those so congenitally severely cognitively impaired as to fall within or below the borderline personhood threshold.

The second implication is that it would be morally unjustifiable to sacrifice great-ape/human chimeras for the sake of human borderline persons, contrary to what Shaw et al. endorsed. This is because great-ape/human chimeras and human borderline persons would have time-relative interests of the same strength. While it would be morally permissible to kill a great-ape/human chimera for saving the life of a human person, according to the level-down position; it would be morally impermissible to kill the chimera for saving the life of a human borderline person and even less for saving the life of a human nonperson.

The third implication is that Shaw et al. would have to accept that instead of waiting a decade, or more, while scientists develop great-ape/human chimeras whose organs we could use for transplantation, we should start using human borderline persons’ organs *today!* By killing and using the organs of human borderline persons and nonpersons right away we could avoid the deaths of large numbers of human persons. Even more so, some of the resources that we would need for the creation and maintenance of the great-ape/human chimeras could be saved, and repurposed for other morally worthy ends, given that human borderline persons and nonpersons occur naturally.

At this point Shaw et al. could try to avoid the level-down problems by relying on a relational account of moral status. They could claim that the relations that such human borderline persons, and nonpersons, have with their relatives and those that care for them (i.e. human persons) in fact either enhance their moral status (Steinbock 2011), or grant them the moral status of a paradigmatic person (Kittay 2005, 2009). Thus while it would be morally permissible to kill great-ape/human chimeras for their organs it would not be morally permissible to kill human borderline persons and nonpersons for their organs. One difficulty with this approach is that it does not automatically entail that we would not be able to harvest organs from *all* humans that are borderline persons or nonpersons. It instead implies that it would only be immoral to harvest organs from humans that are borderline persons or nonpersons and that *have a special relation* with some other person. Thus, all human borderline persons and nonpersons that are *not* in such relationships could in fact be killed for their organs. The second difficulty is that this same conclusion, regarding special relations, would apply to great apes and great-ape/human chimeras. For example, if a person raised, or took care of, a great ape and established with her a significant relation then the moral status of this great ape would be modified to the same degree as that of a borderline human that enters the same type of relationship with another person. This shows that even an appeal to a relational account of moral status entails neither a total prohibition of the killing of human borderline persons and nonpersons for their organs, nor the complete moral acceptability of the killing of great-ape/human chimeras for their human organs.

Shaw et al. could claim that even if human borderline persons were not in relationships that enhanced their moral worth it would be inconceivable to take them out of their homes to be killed for their organs. Even if we accept this, we can still imagine possible scenarios where we could morally obtain and kill such human borderline persons, and nonpersons, for their organs (if the levelling-down approach were correct). For example, we could raise abandoned human borderline persons and nonpersons for such purposes, just as we would raise great-ape/human chimeras, in a special location. It is easy to imagine a location where those that ‘take care’ of such humans do so remotely and without knowing who they are taking care of, and thus do not generate any significant relation with them. If these conditions were met then it would be morally unproblematic to kill such human borderline persons, and nonpersons, for their organs.

The fact that personhood and moral status are species neutral entails that the conclusions that Shaw et al. happily apply to great apes and great-ape/human chimeras would also apply, in principle, to humans that are borderline persons. Confronted with this problem Shaw et al. have either to accept that it is morally acceptable to kill great apes, great-ape/human chimeras and some *human borderline persons* for their organs, or they would have to reject this proposition. I think that the authors would endorse the latter stance.

In this section I have tried to show that a hybrid account of morality can explain why killing borderline persons for their organs is morally inadmissible. If this is correct then it follows that Shaw et al.’s appeal to the subsidiarity and proportionality principles to solve the issue is unwarranted. I have also shown that Shaw et al. failed to realise an important implication of adopting a species neutral stance. This implication is that their conclusions equally apply to human borderline persons and nonpersons (or some human borderline persons and nonpersons, if we endorse a relational account of moral status).

## Final remarks

Is it morally permissible to kill nonhuman-primates/human chimeras’ for their organs? The answer depends on the cognitive capacities that such chimeras possess. On the one hand, if such nonhuman primates have great enough cognitive capacities to classify them as persons, or borderline persons, then the answer is that, in principle, *it is morally impermissible to kill them for their organs*. On the other hand, if they are not persons, or borderline persons, then the answer is that it could be permissible to kill them for their organs. Now, according to a species neutral account this second statement also entails that certain human beings, those that are nonpersons, could be morally sacrificed for their organs (e.g. those unloved humans that are congenitally severely cognitively impaired). Is this correct?

It is what logically follows from the argument. Even if we accepted a relational account of moral status there would most probably be certain human nonpersons that could be morally sacrificed for their organs. It is true that many will find this option appalling. Is there a way to be consistent and at the same time reject the use of unloved human nonpersons as organ sources? I doubt there is to a full extent: even when this might be the case there is still a well-known multipolar option that would widely reduce the need to kill nonpersons (human or otherwise). The multipolar option would entail, among other things, adopting health programmes that promoted healthy lifestyles, a default opt-out donation system, creating incentives for people to autonomously make live organ donations, and substantively investing in research that aimed at the creation of mechanical organs, 3-D printed human organs and in vitro organs using scaffolds. In addition to this multipolar solution we should also further discuss the ethics of making after death organ donation mandatory (Harris 2002). All these efforts would bring down the number of people that would need to rely on killing nonpersons (human or nonhuman) in order to acquire healthy organs.

## Footnotes

1 In this work I use “cognitive capacities”, “mental capacities” and “psychological capacities” interchangeably.

2 Even when it is possible that human organs could be created within other HNH-chimeras, for example elephant/human chimeras or mouse/human chimeras, it is important to notice that the anatomical structure of the human body restricts the ‘type’ of organs that could be used for transplantation.

3 Here ‘HNH-chimera’ should be understood as a chimera predominantly constituted by nonhuman components and with only ‘a few’ human cells.

4 It is important, again, to stress that Shaw et al. do not distinguish between human persons and human nonpersons and thus they only talk about benefits conferred to *humans*.

5 In the following section I discuss how conflict of interests between persons and borderline persons should be resolved.

6 The empirical evidence supporting this claim is cited at length by DeGrazia (1997, 2005, 2007) and Ravelingien et al. (2006).

7 DeGrazia defends that the three great apes that are linguistically competent in fact are fully-fledged persons. In what follows I will not discuss the possibility of linguistically competent great apes or great-ape/human chimeras.
